# Supplementary material for: Waist circumference and low high-density lipoprotein cholesterol as markers of cardiometabolic risk in Kenyan adults
Source: PLoS One. 2021 Feb 25;16(2):e0247600. doi: 10.1371/journal.pone.0247600 (PMC7906307; doi:10.1371/journal.pone.0247600)
Supplement: S1 Table — (DOCX) [file pone.0247600.s001.docx]

| **S1 Table.** Characteristics of adult Kenyans with impaired glucose tolerance (n=196) | | |
| --- | --- | --- |
|  | Mean/n (%) | SD |
|  |  |  |
| Age (years) | 41.8 | 10.8 |
| Female sex | 125 (63.8) |  |
|  |  |  |
| ***Anthropometry and body composition*** |  |  |
| Body mass index (kg/m^2^) | 24.2 | 5.7 |
| Waist circumference (cm) | 85.2 | 14.5 |
| Visceral adipose tissue (cm) | 6.5 | 2.0 |
| Subcutaneous adipose tissue (cm) | 2.0 | 1.4 |
| Hepatic steatosis n (%)^*^ | 30 (33.3) |  |
|  |  |  |
| ***Biochemistry*** |  |  |
| Fasting venous glucose (mmol/L) | 6.1 | 3.5 |
| 2-h blood venous (mmol/L) | 9.1 | 5.1 |
| Fasting serum insulin (pmol/L)^**^ | 26 | 16-48 |
| Fasting total plasma Cholesterol (mmol/L) | 4.1 | 1.2 |
| Fasting plasma HDL (mmol/L) | 1.1 | 0.4 |
| Fasting plasma Total cholesterol/HDL-C ratio^**^ | 3.9 | 3.0-4.8 |
| Fasting plasma LDL (mmol/L) | 2.5 | 0.9 |
| Fasting plasma VLDL (mmol/L) | 0.6 | 0.4 |
| Fasting plasma Triglyceride (mmol/L)^**^ | 0.9 | 0.7-1.4 |
|  |  |  |
| ***Blood pressure (mmHg)*** |  |  |
| Systolic | 124.4 | 19.9 |
| Diastolic | 76.9 | 13.1 |
| Abbreviations: HDL: high-density lipoprotein cholesterol, LDL: low-density lipoprotein cholesterol, VLDL: very low-density lipoprotein cholesterol, SD: standard deviation  ^*^Based on ultrasound scanning semi-quantitative liver fat score (n=90)  ^**^Median (interquartile range) | | |
