# Supplementary material for: Waist circumference and low high-density lipoprotein cholesterol as markers of cardiometabolic risk in Kenyan adults
Source: PLoS One. 2021 Feb 25;16(2):e0247600. doi: 10.1371/journal.pone.0247600 (PMC7906307; doi:10.1371/journal.pone.0247600)
Supplement: S2 Table — (DOCX) [file pone.0247600.s002.docx]

| **S2 Table.** Association between high-density lipoprotein cholesterol (HDL) and waist circumference with body composition, biochemistry, and blood pressure in adult Kenyans with impaired glucose tolerance (n=196) | | |
| --- | --- | --- |
|  | HDL | Waist circumference |
| **Dependent variable** | **B (95 % CI)** | **B (95 % CI)** |
| Visceral adipose tissue (cm) | -0.7 (-1.4; -0.03) ^*^ | 0.1 (0.1; 0.1) ^*^ |
| Subcutaneous adipose tissue (cm) | 0.2 (-0.3; 0.7) | 0.1 (0.1; 0.1) ^*^ |
| Fasting venous glucose (mmol/L) | -0.1 (-1.5; 1.2) | 0.05 (0.01; 0.1) ^*^ |
| 2-h venous glucose (mmol/L) | 0.02 (-2.0; 2.0) | 0.05 (-0.01; 0.1) |
| Low-density lipoprotein cholesterol (mmol/L) | 0.4 (0.1; 0.8) ^*^ | 0.02 (0.01; 0.03) ^*^ |
| Total cholesterol (mmol/L) | 1.3 (0.9; 1.7) ^*^ | 0.02 (0.01; 0.04) ^*^ |
| Systolic blood pressure (mmHg) | 9.5 (2.4; 16.6) ^*^ | 0.5 (0.3; 0.7) ^*^ |
| Diastolic blood pressure (mmHg) | 4.5 (-0.4; 9.4) | 0.3 (0.2; 0.5) ^*^ |
| **Dependent variable (log transformed)** | **e^B^ (95 % CI)** | **e^B^ (95 % CI)** |
| Triglyceride (mmol/L) | 0.80 (0.67; 0.96) ^*^ | 1.01 (1.01; 1.02) ^*^ |
| Fasting serum insulin (pmol/L) | 1.13 (0.83; 1.53) | 1.03 (1.02; 1.04) ^*^ |
| Data are linear regression analyses adjusted for age and sex. e^B^ denotes a back-transformed coefficient and interpreted as a ratio. ^*^ p<0.05 | | |
